# Reliability of Xsens inertial measurement unit in measuring trunk accelerations: a sex-based differences study during incremental treadmill running

**DOI:** 10.3389/fspor.2024.1357353

**Published:** 2024-03-27

**Authors:** Unai Miqueleiz, Roberto Aguado-Jimenez, Pablo Lecumberri, Ibai Garcia-Tabar, Esteban M. Gorostiaga

**Affiliations:** ^1^ Department of Health Sciences, Public University of Navarra, Pamplona, Spain; ^2^ Studies, Research and Sports Medicine Centre (CEIMD), Government of Navarre, Pamplona, Spain; ^3^ Department of Mathematics, Public University of Navarre, Pamplona, Spain; ^4^ Society, Sports and Physical Exercise Research Group (GIKAFIT), Department of Physical Education and Sport, Faculty of Education and Sport, University of the Basque Country, Vitoria-Gasteiz, Spain

**Keywords:** inertial sensor, biomechanics, treadmill running, wearable, endurance athletes

## Abstract

**Introduction:**

Inertial measurement units (IMUs) are utilized to measure trunk acceleration variables related to both running performances and rehabilitation purposes. This study examined both the reliability and sex-based differences of these variables during an incremental treadmill running test.

**Methods:**

Eighteen endurance runners performed a test–retest on different days, and 30 runners (15 females) were recruited to analyze sex-based differences. Mediolateral (ML) and vertical (VT) trunk displacement and root mean square (RMS) accelerations were analyzed at 9, 15, and 21 km·h^−1^.

**Results:**

No significant differences were found between test-retests [effect size (ES)<0.50)]. Higher intraclass correlation coefficients (ICCs) were found in the trunk displacement (0.85-0.96) compared to the RMS-based variables (0.71–0.94). Male runners showed greater VT displacement (ES = 0.90–1.0), while female runners displayed greater ML displacement, RMS ML and anteroposterior (AP), and resultant euclidean scalar (RES) (ES = 0.83–1.9).

**Discussion:**

The IMU was found reliable for the analysis of the studied trunk acceleration-based variables. This is the first study that reports different results concerning acceleration (RMS) and trunk displacement variables for a same axis in the analysis of sex-based differences.

## Introduction

Advances in technology during the last decades have potentiated interest in running biomechanics. Measuring instruments have evolved from frame-after-frame photography to high-speed cameras and force platforms as gold standards (1). To mitigate the inaccessibility to these gold standard instruments, other devices such as inertial measurement units (IMUs) were developed, and this technology recently looks more promising for the study of running biomechanics (2). The advantages of using the IMU include both the portability out of the laboratory allowing objective analyses in real settings and the affordable price compared to the gold standard instrumentation (3). IMU development, therefore, helps progress toward instrumentation accessibility (4). This technology contains a triaxial accelerometer, gyroscope, and magnetometer that capture data in three axes to allow a comprehensive assessment of human locomotion and the determination of the running gait phases (5). The data recorded with IMUs is then usually converted into metrics for a wide range of purposes. For example, Xsens technology IMU instrumentation has been previously used to study knee flexion angles and ground reaction forces during human locomotion in healthy populations (6, 7) and spatiotemporal responses to gait in patients with different pathologies (8, 9). Previous research also used this technology for injury rehabilitation and running and sprint performance purposes (10–13).

There is a wide range of IMU-based variables that could be calculated with commercially available software. Among these, spatiotemporal and kinetic variables have been mainly studied and confronted against gold standard instruments (14–16). However, other important variables, such as the vertical (VT) and mediolateral (ML) trunk displacements and the resultant Euclidean scalar (RES) variable, are understudied to date. The latter is usually calculated from the root mean square (RMS) for each axis and represents the overall magnitude of body acceleration, a biomechanical variable related to oxygen uptake and thus to energy expenditure (17). VT displacement has been traditionally measured with optoelectronic motion analysis systems or accelerometers during either overground or treadmill running (12, 18–24). ML displacement seems to be related to the energetic cost of running and strain rates (25–27). However, studies analyzing ML displacement are scarce (26). Furthermore, previous studies found a strong relationship between RES and oxygen uptake during treadmill locomotion (17). These RMS and RES variables were found to be valid and reliable during treadmill running compared with optimal motion capture as a reference system (28). Since the applicability of these IMU variables is dependent on their measurement reliability, it is deemed necessary to further study the repeatability of these measurements before implementation (29). The reporting of these data would enable a fair and correct clinical interpretation over time and, therefore, might widespread the utilization of these variables for running assessments.

Most IMU biomechanical running performance analyses in runners are carried out on men. Nevertheless, it is expected that sex differences would be apparent in the trunk displacements, which could explain differences in the energy expenditure in running (30). To our knowledge, there is only one study that has found greater ML accelerations in female runners measured at one single speed (31). There is, therefore, a lack of research on sex-based differences concerning trunk displacements and accelerations during running at different speeds.

Therefore, the present study aimed to (i) evaluate the reliability of both trunk displacements and RMS-RES variables measured with an IMU device and (ii) analyze the sex-based differences concerning these variables in a group of endurance runners during an incremental treadmill running test. The results of this study might interest runners, coaches, and practitioners for a better understanding of variables that could be related to both running economy and performance.

## Materials and methods

### Participants

A total sample of 30 endurance runners (37.0 ± 9.7 years) participated in this investigation. The Clinical Research Ethics Committee of the Local Institutional Review Board (PI/012-20) approved this study according to the Declaration of Helsinki. All participants signed the informed consent as required. This investigation consisted of two-part complementary studies performed within a month.

#### Study I (reliability study; *n* = 18)

The study was conducted on 13 male and 5 female runners (body mass, 65.3 ± 8.9 kg; height, 174 ± 7.0 cm). The runners completed a test–retest design protocol separated by 7–10 days. This study aimed to analyze the smallest amount of change outside the error of the instrument used.

#### Study II (sex-based differences study; *n* = 30)

The study was conducted with 18 runners from Study I, in addition to 12 participants. The sample was therefore composed of 15 male [body mass: 69.5 ± 6.6 kg; height: 177 ± 4.5 cm; maximal estimated aerobic speed (MAS): 18.6 ± 1.4 km·h^−1^] and 15 female runners (body mass, 53.7 ± 4.6 kg; height, 164 ± 3.2 cm; MAS, 17.1 ± 1.3 km·h^−1^). This study aimed to analyze the biomechanical differences between sexes in a mixed group of endurance runners.

The participants were familiarised with the procedures of this study because they have regularly performed several treadmill tests in our laboratory for training or health purposes in the previous years. The inclusion criteria required participants to run >2 days per week over the last year. Any injury in the previous 3 months before the beginning of the study was considered an exclusion criterion. The tests required a rested state in the absence of alcohol and caffeine intake in the previous 24 h. The participants were asked to wear their usual running shoes and the same ones during both tests. Laboratory conditions were maintained within standard conditions (20°C temperature/27% humidity).

Each testing session was performed on a motorized treadmill (ERG-ELEK-EG4, ISSA Engineering, Vitoria, Spain). The testing protocol consisted of a standardized warm-up running for 6 min, followed by 3 min of rest preceding a maximal incremental continuous running test to exhaustion. The treadmill slope was 1%, and the initial speed was 8 km·h^−1^ and was incremented by 1 km·h^−1^ every min (32). Finally, 5 min after the end of the incremental test, the participants ran on the treadmill for 30 s at 21 km·h^−1^.

Body mass and height were measured in each subject on a leveled platform scale (Seca 700, Seca Corp., Hamburg, Germany) prior to the IMU colocation (MTw, 3DOF Human Orientation Tracker, Xsens Technologies BV, Enschede, the Netherlands). One IMU was fastened at lumbar spine level [L4–L5; center of mass acceleration (33)], which allowed us to accurately determine both spatiotemporal (34) and kinematic parameters (35) during running. Heart rate (HR) (Polar Electro Oy, M400 watch and H7 strap, Kempele, Finland) was monitored during the tests, and maximal HR (HR_max_) was established as the highest HR recorded. The MAS was calculated during the maximal incremental test using the formula: MAS = Sf + (*t*/60), where Sf is the last completed speed in km·h^−1^ and *t* is the time in seconds of the uncompleted stage.

VT and ML trunk displacements (cm) were calculated by double integration of the corresponding acceleration. A polynomial estimation of the baseline removed drift errors in speed (first integration) and displacement (second integration) was performed. Displacement was established as the difference between the averages of the points from one extreme to the other during the recording. RES was calculated from the RMS of each axis (m·s^−2^), which corresponds to the area of the square of the signal per unit of time with a scaling factor (17).

### Data processing and analysis

Acceleration and orientation signals were recorded and analyzed with a proprietary software (Movalsys, Navarre, Spain). Data transformation and further analyses were implemented with MatLab R2016a (MathWorks Inc.; Natick, MA, USA). A fusion algorithm computes a 3D space orientation of the sensor-fixed reference frame S with respect to an Earth-fixed reference frame E. The analysis requires the acceleration component in the forward direction of the run, rotating frame E so that the *y*-axis points heading direction. The sampling rate was set at 120 Hz, and a 25 Hz low-pass filter with a zero-lag Butterworth was applied. Twenty seconds at each speed was analyzed to ensure a minimum of 10 strides per speed (36). The data of participants on the first day from “Study I” were used for “Study II.”

### Statistical analysis

Mean and standard deviation (SD) were calculated for each measure. The data were screened for normality of distribution and homogeneity of variances using the Shapiro–Wilk and Levene's tests, respectively, and when appropriate sphericity (Mauchly's test). Paired *t*-test and mean bias ± limits of agreement (LoAs) based on *t* distribution with n-1 degrees of freedom (Bland and Altman plots) were used to analyze differences between the two testing sessions (“Study I”). Linear regressions from Bland and Altman plots (Appendix I) were calculated to study the systematic error in the distribution of the data points. One-way analysis of variance (ANOVA) with Bonferroni's adjustment was performed to detect differences among speeds. The reliability analyses included the following calculations: The intraclass correlation coefficient (ICC) (2,1), two-way mixed-effects model and absolute agreement with 95% confidence intervals (CI); the standard error of measurement (SEM) of paired data (SEM = SD_diff_/√2), the coefficient of variation [CV = (SEM/mean)*100]; and the minimal detectable change (MDC = SEM × 1.65 ×√ 2). ICC values between 0.5 and 0.75 indicated *moderate* reliability, between 0.75 and 0.9 indicated *good* reliability, and >0.9 indicated *excellent* reliability (37). CV was classified as *trivial* (CV ≤ 5%), *small* (5%<CV ≤ 10%), and *moderate* (CV > 10%) (38). Means, CV, and MDC were calculated for 9, 15, and 21 km·h^−1^ to ensure the inclusion of all 18 participants. In “Study II,” the differences were evaluated by two-factor repeated-measures ANOVA with Bonferroni’s corrections. The differences (e.g., ESs) were interpreted using previously suggested thresholds: *small* (0.2), *moderate* (0.6), *large* (1.2), and *very large* (2.0) (39). Significance was set at *p *< 0.05 for analyses not requiring *post hoc* adjustment. A statistical analysis was performed with SPSS version 20.0 (SPSS Inc. Chicago, USA).

## Results

### Study I (reliability study)

During the maximal incremental running test, no significant differences were found between both testing days in HR_max_ [183 ± 13 vs. 181 ± 13 beats·min^−1^; *p *= 0.16; effect size (ES) = 0.15, *small*] or MAS (18 ± 1.9 vs. 17.8 ± 1.7 km·h^−1^; *p *= 0.26; ES = 0.11, *small*). The mean absolute values of the IMU variables studied for the selected speeds at each of the test days are shown in Table 1. No significant differences were observed between the first and second tests in terms of trunk displacements and RMS-RES (*p *= 0.22; trunk displacements, ES < 0.14, *small*; RMS-RES, ES < 0.49, *small*). Bland and Altman analyses between both tests for each variable at any speed showed the presence of non-significant systematic errors (*p *= 0.08–0.99; *R*^2 ^< 0.07; mean bias, −0.33–0.21) (Appendix I). ANOVA analyses showed the presence of random errors in VT displacement (0.43 cm), ML displacement (0.22 cm), RMS ML (0.47 m·s^−2^), RMS anteroposterior (AP) (0.49 m·s^−2^), RMS VT (0.63 m·s^−2^), and RES (0.74 m·s^−2^). *Good* to *excellent* ICC values were observed both for the trunk displacement variables (0.85–0.96) and the RMS-RES (0.71–0.94), while all the CVs at 15 and 21 km·h^−1^ were *trivial* in the trunk displacements. The CVs in RMS ML and AP were *small* to *trivial* and were *trivial* in RMS VT. RES showed the lowest CV among all the measured variables. ANOVA analyses indicated that ICCs increased between 9 and 21 km·h^−1^ (*p *= 0.001; ES > 3.1, *very large*), while CVs remained similar (*p *= 0.09) (Table 2).

**Table 1:** Trunk displacements and RMS-RES variables analyzed at the selected speeds (*n* = 18).

Variable	9 km·h^−1^		15 km·h^−1^		21 km·h^−1^	
	Session 1	Session 2	Session 1	Session 2	Session 1	Session 2
VT displ. (cm)	10.2 ± 1.78	10.0 ± 1.67	8.49 ± 1.43^a^	8.58 ± 1.40^a^	6.12 ± 1.46^a^^,^^b^	6.31 ± 1.35^a^^,^^b^
ML displ. (cm)	2.44 ± 0.81	2.43 ± 0.82	4.52 ± 1.36^a^	4.45 ± 1.35^a^	5.21 ± 1.46^a^	5.27 ± 1.54^a^
RMS ML (m·s^−2^)	4.0 ± 1.0	4.0 ± 1.1	6.1 ± 1.4^a^	6.0 ± 1.2^a^	8.9 ± 1.7^a^^,^^b^	9.3 ± 2.6^a^^,^^b^
RMS AP (m·s^−2^)	4.8 ± 0.80	4.7 ± 0.80	8.3 ± 1.4^a^	7.7 ± 1.0^a^	11.7 ± 2.4^a^^,^^b^	11.5 ± 1.9^a^^,^^b^
RMS VT (m·s^−2^)	11.7 ± 1.4	11.6 ± 1.4	12.3 ± 1.5	12.3 ± 1.4	12.7 ± 2.2	12.5 ± 2.0
RES (m·s^−2^)	13.3 ± 1.6	13.2 ± 1.5	16.2 ± 1.7^a^	15.8 ± 1.4^a^	19.5 ± 3.1^a^^,^^b^	19.5 ± 3.1^a^^,^^b^

Data presented as mean ± SD. VT, vertical; ML, mediolateral; AP, anteroposterior; RMS, root mean square; RES, resultant Euclidean scalar. Significant differences.

^a^ Different from 9 km·h^−1^.

^b^ Different from 15 km·h^−1^.

**Table 2:** Reliability data of the IMU variables analyzed at the selected speeds (*n* = 18).

Variable	9 km·h^−1^		15 km·h^−1^		21 km·h^−1^		Mean bias (9–21 km·h^−1^)	LoA (9–21 km·h^−1^)	*R*^2^ (*p*) (9–21 km·h^−1^)	CV% (9/15/21 km·h^−1^)	MDC (9/15/21 km·h^−1^)
	ICC	95% CI	ICC	95% CI	ICC	95% CI					
VT displ. (cm)	0.85	(0.66–0.94)	0.91	(0.78–0.96)	0.94	(0.84–0.98)	−0.08–0.21	(−1.4–1.4)	0.06 (>0.05)	4.2/3.2/3.4	0.99/0.64/0.49
ML displ. (cm)	0.85	(0.63–0.94)	0.96	(0.89–0.98)	0.94	(0.83–0.98)	−0.07–0.00	(−0.95–0.88)	0.00 (>0.05)	6.4/4.2/4.1	0.36/0.44/0.50
RMS ML (m·s^−2^)	0.86	(0.66–0.95)	0.83	(0.60–0.93)	0.92	(0.79–0.97)	−0.13–0.01	(−1.4–1.3)	0.00 (>0.05)	6.0/6.0/2.7	0.56/0.84/0.58
RMS AP (m·s^−2^)	0.82	(0.58–0.93)	0.71	(0.22–0.89)	0.90	(0.75–0.96)	−0.32 to −0.10	(−1.5–0.96)	0.01 (>0.05)	4.6/5.5/4.9	0.51/1.0/1.3
RMS VT (m·s^−2^)	0.74	(0.44–0.89)	0.88	(0.71–0.95)	0.94	(0.82–0.98)	−0.25 to −0.13	(−1.9–1.5)	0.04 (>0.05)	4.9/2.6/3.1	1.3/0.75/0.91
RES (m·s^−2^)	0.77	(0.49–0.91)	0.81	(0.57–0.93)	0.96	(0.88–0.98)	−0.33 to −0.14	(−2.1–1.5)	0.06 (>0.05)	4.0/2.5/2.1	1.2/0.93/0.95

ICC, intraclass correlation coefficient; CI, confidence interval; LoA, limit of agreement; CV, coefficient of variation; MDC, minimal detectable change; VT, vertical; ML, mediolateral; AP, anteroposterior; RMS, root mean square; RES, resultant Euclidean scalar. ICC correlation magnitudes were all significant (*p* < 0.001).

The ANOVA results showed that as the speed increased, there was a decrease in VT displacement (*p *= 0.007; *r* = −0.74; ES = 1.0–2.5, *moderate* to *very large*) and an increase in ML displacement (*p *< 0.001; *r* = 0.68; ES = 1.8–2.3, *large* to *very large*)*.* RMS increased with increasing speed in the ML and AP axes (*p *< 0.001; *r* = 0.79 and 0.90, respectively; ES = 1.3–4.6, *large* to *very large*) but remained unchanged in the VT axis (*p *= 0.23; *r* = 0.24; ES < 0.54, *small*). RES values increased with increasing speed (*p *= 0.002; *r* = 0.77; ES = 1.4–2.6, *large* to *very large*).

### Study II (sex-based difference study)

**Figure 1:**
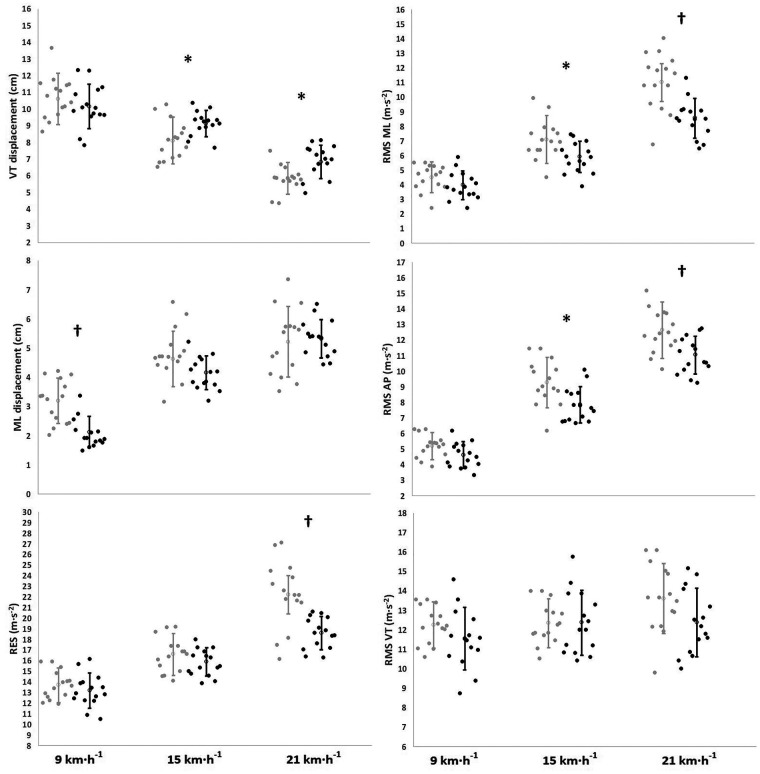
Sex-based differences in the measured IMU-based variables as a function of running speed (*n* = 30). The empty circles and error bars denote mean ± SD, respectively. The dots are individual values. The black dots denote the male runners. The gray dots denote the female runners. VT, vertical; ML, mediolateral; AP, anteroposterior; RMS, root mean square; RES, resultant Euclidean scalar. Significant differences between sexes: * < 0.05; † < 0.01.

The male runners showed greater body mass (*p *< 0.001; ES = 2.8, *very large*), height (*p *< 0.001; ES = 3.4; *very large*), and MAS (*p *= 0.015; ES = 1.1, *moderate*) than those of the female runners. Absolute IMUs trunk both displacement and acceleration for both male and female runners as shown in Figure 1. The male runners had greater VT displacements at both 15 and 21 km·h^−1^ speeds (Δ%* *= 11.1–14.6%; *p *= 0.024–0.037; ES = 0.90–1.0, *moderate*). In contrast, the female runners showed greater ML displacement at 9 km·h^−1^ (Δ%* *= 33.4%; *p *= 0.001; ES = 1.6, *large*), as well as both RMS ML and RMS AP at both 15 and 21 km·h^−1^ speeds (Δ%* *= 15.–6.1%; *p *= 0.005–0.048; ES = 0.83–1.9, *moderate* to *large*), and greater RES at 21 km·h^−1^ (Δ%* *= 16.3%; *p *= 0.006; ES* *= 1.2, *very large*). No significant differences were found in RMS VT between the male and female runners (*p *= 0.14–0.96). The percentages of differences between sexes were all greater than the MDC reported (Table 2).

## Discussion

The purposes of this study were to determine the reliability of both trunk displacements and RMS-RES variables measured with an MTw IMU using a test–retest design, considering the inherent biological variability arising from the participants, and to analyze the sex-based differences in these variables in endurance runners while running on a treadmill at incremental speeds. The main finding was that MTw IMU showed a *good* to *excellent* relative reliability and a CV < 4.5% as absolute reliability in the trunk displacements variables and *moderate* to *excellent* relative reliability and a CV < 4.2% as absolute reliability in the RMS-RES variables. In addition, the male runners showed greater VT displacements and lower ML displacement, greater RMS in both ML and AP axes, and lower RES than female runners along the speeds tested. This suggests that there are sex differences in the running patterns in terms of trunk displacements and the magnitude of body accelerations, which according to previous studies could explain differences in running performance (40) and injury incidence (41).

### Study I (reliability study)

VT and ML displacements showed *good* to *excellent* reliability according to the high ICCs (range, 0.85–0.96) and relatively *small* CVs (<6.5%). The reliability was slightly higher in VT (CV < 4.3%) than in ML (CV < 6.5%) and was greater at higher speeds. Highly regular and reliable VT displacement could partially be explained by the fact that the VT direction of run is continuously constrained to gravity (19), and thus, less subjected to system noise than the ML displacement. According to the MDC (Table 2), a runner's change after an intervention should be considered significant if it exceeds 10% (VT) or 15% (ML) of the baseline values at 9 km·h^−1^ and 8% (VT) or 10% (ML) at 21 km·h^−1^.

The RMS-RES variables showed *moderate* to *excellent* reliability and a *small* error (ICCs range, 0.74–0.94; CV < 6.1%). These values were similar in magnitude to those observed in the trunk displacements and were slightly more reliable in RES (CV < 4%) and RMS VT (CV < 5%) than in RMS AP or ML (CV < 6%). The reliability values presented here are inferior to those previously reported for male endurance athletes running on a treadmill (17). However, these authors calculated a single CV for all the speeds tested together (between 2 and 16 km·h^−1^). This differs from the present study in that reliability was calculated at each speed and found to vary across the different speeds. Moreover, a previous study showed higher reliability values than our study in a mixed-sex group of college-aged subjects running on a treadmill (28). However, their subjects performed both tests on the same day, separated by 60–180 s, whereas in our study both tests were separated by 7–10 days. In our study, a longer test–retest delay may increase within-subject random or systematic variation, potentially worsening reliability values compared to the within-day variability assessed (28). Based on the MDC (Table 2), the smallest amount of change that may reflect a true or relevant change in the economy-based variables after an intervention varies from 9 to 14% at 9 km·h^−1^ and from 6 to 11% at 21 km·h^−1^. RES needs the lowest amount of change to detect relevant changes (<6%). The results indicate that the MTw IMU is as reliable as other accelerometry-based devices and it could be used to analyze changes over time, considering the error reported in this study.

### Study II (sex-based difference study)

In the present study, VT values significantly decreased with higher speeds, from 10.2 cm at 9 km·h^−1^ to 6.8 cm at 21 km·h^−1^. These VT values agree with other studies conducted in mixed-sex groups and male recreational runners (12, 19, 22, 31) and international-level male athletes during treadmill or overground running (12, 19, 20, 22, 42), in which VT values were measured using optoelectronic motion analysis, sacral accelerometers, or a single digital video camera (12, 19, 20, 22, 42). The lower VT displacement in female runners could indicate lower VT loading rates and mechanical power per step but also per minute of running (20). This lower VT displacement is suggestive of decreased VT peak force (43), lower extremity joint energy absorption (22), impact shock when the foot hits the ground, and increased running economy (19, 22, 44).

ML displacement increased with increasing speeds in both groups, from average values of 2.1 cm at 9 km·h^−1^ to 5.3 cm at 21 km·h^−1^. However, females had higher ML displacement than males at the lowest speed (9 km·h^−1^). The higher ML displacement, combined with the lack of differences observed in VT displacements between sexes at this speed (Figure 1), and the similar stride frequencies between sexes found in previous studies during running at low speeds (45–47) suggest that females exhibited greater lateral oscillations and greater trunk motion per step and per time unit compared to males. However, at 15 and 21 km·h^−1^, the ML displacements were similar between sexes as shown in Figure 1, but females had lower VT displacements than males. It is suggested that contrary to what was found at the slowest speed, females exhibited less trunk motion per step than males at higher speeds.

On the other hand, RES values significantly increased with higher speeds, ranging from 13.2 m·s^−2^ at 9 km·h^−1^ to 22.2 m·s^−2^ at 21 km·h^−1^. The RES values agree with a study conducted on healthy college-aged individuals of mixed sex who run on a treadmill while wearing an accelerometer placed in line with the top iliac crest (28). However, they disagree with the values previously reported by the same research group in a study performed with male runners measured during treadmill running using the same accelerometer (17). A potential explanation for this inconsistency could be attributed to the methodology. Their formula did not include the number of samples in the dataset inside the square root to calculate RMS. The formula was rectified in their subsequent study which is consistent with our findings (28). In addition, the observed increase in absolute RES with higher speeds has also been observed by others (42). This increase is predominantly due to an increase in the AP (143%) and the ML (145%) axes, particularly in females, rather than in the VT (9%) axis (Figure 1), which suggests an increase in energy expenditure (17). This may explain why the female runners showed higher RES (Figure 1) than male runners. Previous studies also found higher RMS ML in female runners during treadmill running at different speeds between sexes (11.1 vs. 9.6 km·h^−1^) (31). The higher RMS ML may be translated into higher ML loading rates during ground contact which are not useful for propulsion (25) and may result in both an increase in energy expenditure and a decline in running economy. This is also supported by another study (48), which hypothesized that female runners present a reduced neuromuscular control in the musculature of the sagittal plane to decelerate the body during the stance phase and rely more on the frontal plane. However, the novel results of this study suggest the presence of acceleration components that increase the signal power with increasing speed but do not contribute to a higher range of trunk motion (e.g., ML displacements at 15–21 km·h^−1^) (Figure 1). This would mean that trunk displacements alone could not allow general conclusions between sexes in running gait and either some spatiotemporal and/or kinetic variables should be brought into the equation. Thus, caution should be taken when runners are not assessed using all the spectrum variables measured by the IMUs.

Some limitations need to be addressed. First, the incremental running protocol considered speeds up to 21 km·h^−1^ during treadmill running. Thus, the present findings are likely to apply to the speeds and running surface used. Second, accelerations were analyzed for 20-s at each speed. These 20-s were considered sufficient to ensure the analysis of a minimum of strides in all participants. However, the higher average number of steps analyzed at 21 km·h^−1^ (−70) compared with 9 km·h^−1^ (−54) could somehow explain the better reliability found at higher speeds. Third, the implication of changes in signal power which do not imply changes in the trunk motion at a certain axis needs further study in terms of both performance and running-related injuries.

## Conclusions

The findings of this study demonstrated that the measurement of trunk displacements and RMS-RES variables with the MTw IMU had a moderate to excellent degree of test–retest reliability in endurance runners while running on a treadmill. Furthermore, this study indicates that the higher the speed, the better the reliability. Another novel aspect of this study is the reported explicit error of measurement (CV and MDC). These reported values allow us to analyze whether an intervention implies a relevant clinical change of these kinematic and energy-based variables measured by an MTw IMU. On the other hand, female runners seem to show lower VT displacements at higher speeds. These variables alone did not explain the higher RES in female runners, but accelerations (RMS) in both ML and AP axes could shed some light on sex differences in running performance. This study reports promising results that are of very great interest to runners, coaches, physiotherapists, and practitioners in understanding how these variables, which may be related to running economy (19) and injury incidence (41), are performed according to sex and speed.

## Data Availability

The original contributions presented in the study are included in the article/Supplementary Material, and further inquiries can be directed to the corresponding author.
